# Remote Monitoring of Cardiac Devices and Clinical Outcomes in Patients with Structural Heart Diseases: Rationale and Design of the ReVe Study

**DOI:** 10.3390/jcm14041150

**Published:** 2025-02-11

**Authors:** You-Mi Hwang, Sung-Won Jang

**Affiliations:** 1 Department of Cardiology, St. Vincent’s Hospital, The Catholic University of Korea, Seoul 16247, Republic of Korea; youmi0607@naver.com; 2 Catholic Research Institute for Intractable Cardiovascular Disease (CRID), College of Medicine, The Catholic University of Korea, Seoul 06591, Republic of Korea; 3 Cardiovascular Center, Eunpyeong St. Mary’s Hospital, The Catholic University of Korea, Seoul 03312, Republic of Korea

**Keywords:** artificial, cardiac resynchronisation therapy devices, defibrillators, implantable, remote monitoring

## Abstract

**Background/Objectives:** Whether remote monitoring reduces mortality in patients with heart failure remains controversial, and research on remote monitoring in South Korea is generally lacking. Therefore, we aim to evaluate the safety and efficacy of remote monitoring for patients in South Korea with severe structural heart diseases who have an implantable cardioverter–defibrillator or cardiac resynchronisation therapy pacemaker/defibrillator. **Methods:** This ReVe study is a multicentre, prospective, observational cohort study in which we will comprehensively evaluate the impact of remote monitoring on cardiovascular-related death and hospital admissions related to pre-existing cardiovascular disease (primary outcomes) and satisfaction with and cost of remote monitoring and the healthcare provider workload (secondary outcomes). Two patient groups are being recruited: (1) Patients in the historical group (*n* = 225) already had a cardiac implantable electronic device implanted after January 2020 and have attended outpatient device check-ups. (2) Those in the initiating group (planned *n* = 225) will undergo cardiac implantable electronic device implantation during this study. In-office visits are scheduled for every 3–6 months. The time/medical cost efficiency and satisfaction index will be evaluated during the 24-month follow-up period. Questionnaires regarding patient satisfaction will be administered every 6 months. **Conclusions:** This is the first prospective study involving patients with structural heart diseases who have implanted high-power cardiac electronic devices. It will provide insights into remote monitoring applications in South Korea and evidence for their use in such patients. It will also provide evidence of the efficacy, safety, and satisfaction with remote monitoring in this population.

## 1. Introduction

Although reports from Western countries have indicated the effectiveness of wireless remote monitoring (RM) of implantable cardiac devices in reducing hospitalisation rates and early diagnosis of disease exacerbation among patients with severe cardiac diseases, such as those who have undergone resynchronisation therapy or defibrillator implantation, its impact on improving survival rates remains debatable [1,2,3]. However, among patients with structural heart disease, continuous wireless monitoring with an implanted cardiac device can not only provide psychological reassurance for both the patient and healthcare providers but also improve the early detection and management of disease progression, which may lead to a reduction in hospitalisation rates and an improvement in prognosis [4]. Therefore, research related to remote monitoring has gained interest, as it may help meet expectations for continued clinical improvements.

Therefore, we designed this study to evaluate the clinical stability and cost-effectiveness of RM in individuals implanted with high-voltage devices who were diagnosed with structural heart disease (such as cardiomyopathies, heart failure with reduced left ventricular ejection fraction [LVEF; below 40%], or congenital heart disease with heart failure) via cardiac ultrasound or computed tomography/magnetic resonance imaging.

South Korea has a unique healthcare system characterised by excellent medical accessibility and well-established healthcare insurance, ensuring that most citizens have access to high-quality medical services [5,6]. The national health insurance system in South Korea offers substantial benefits for patients with severe illnesses. Consequently, for those with heart disease, the out-of-pocket costs are lower than those in other countries, and access to healthcare is exceptionally good. However, the consequence of this national insurance system is that patients are concentrated in university hospitals or other large, tertiary-level hospitals that have high manpower and mobility. In this context, telemedicine is an important aspect of personnel training, which may exacerbate the concentration of patients and the profit gap between hospitals. Therefore, compared with Western countries, South Korea has faced delays in insurance applications and the domestic introduction of new medical technologies and treatments.

As the demand for telemedicine has increased since the coronavirus disease 2019 pandemic, interest in telemedicine for various conditions has also continued to grow [7,8,9,10]. The introduction of RM services for cardiac implantable electronic devices (CIEDs) that had already been validated was accelerated during the lockdown, and their safety has been demonstrated abroad [11,12]. However, domestic medical experts have little experience with RM, which is still not commonly used in primary, secondary, and tertiary medical centres. We reported the first pilot study (the remote care study) on the efficacy, cost-effectiveness, and safety of RM of implantable cardiac devices in Korea [13,14,15]. We noted that RM yielded time-saving benefits with advanced care to the study participants without increasing the rate of adverse clinical events; however, it also increased the clinicians’ workload. The remote care study had a small sample comprising patients without structural heart diseases and pacemakers. Therefore, we could not elucidate the efficacy and clinical safety of RM for patients with severe structural heart diseases. In line with our previous study, we designed a clinical trial to evaluate the hospitalisation rate, cardiocerebrovascular events, cardiac death, and socio-economic efficacy of RM in two groups of patients implanted with high-power devices with severe structural heart diseases. We also aim to provide evidence for the cost and personnel allocation for RM, along with efficiency and patient satisfaction with RM care within the domestic healthcare system.

## 2. Materials and Methods

### 2.1. Study Design

The RemoteVerify (ReVe) study is a multicentre, prospective, observational study that will be conducted at eight university hospitals (St. Vincent’s Hospital, The Catholic University of Korea; Eunpyeong St. Mary’s Hospital, The Catholic University of Korea; Seoul St. Mary’s Hospital, The Catholic University of Korea; Keimyung University Hospital; Kyungpook National University Hospital; Pusan National University Hospital; Yeungnam University Medical Center; Pusan National University Yangsan Hospital) in Korea, targeting 450 patients (each centre will enrol 50 to 70 patients). This sample size was based on the assumption that the 2-year incidence of major adverse cardiac and cerebrovascular events of patients with structural heart diseases is 10% to 15%, with a confidence interval of 95% and a margin of error of 5%. The desired sample size was calculated as 139 to 196 participants per group using G*Power version 3.1.9.7 [16], and similar patients are needed in the two groups that differ in the timing of RM application. Considering an anticipated 10% loss to follow-up during the study period, the total number of participants needed for this study was determined as 442. We rounded this number up to 225 per group. An interim analysis will be conducted using the variables collected by the planned patient enrolment period (24 months). A second interim analysis will be conducted after at least 12 months of follow-up data have been collected. The final analysis will be performed at the study endpoint after all participants have completed the 24-month follow-up. Starting from the interim analysis, subgroup analyses will be conducted based on the type of underlying heart disease (hypertrophic cardiomyopathy/ischaemic cardiomyopathy/dilated cardiomyopathy/others) and the left ventricular ejection fraction (LVEF; <30%, 30–45%, >45%).

Eligible study participants will need to meet the following criteria: patients (1) aged 20 years or older with (2) an LVEF below 40% or a structural heart disease confirmed via cardiac imaging, (3) an implantable cardioverter–defibrillator (ICD) or a cardiac resynchronisation therapy (CRT) device compatible with Biotronik Ltd. (Berlin, Germany) wireless monitoring devices, and (4) a predicted life expectancy of more than 6 months, (5) capable of understanding and completing the simple consent form, and (6) never having been enrolled in clinical trials that involve RM before. Patients will be informed that if they want to withdraw their consent to this study, they can stop participating in this clinical trial, and their data will be collected until the withdrawal date. Exclusion criteria are as follows: (1) patients with a life expectancy of 6 months or less, (2) patients with cognitive impairment who are unable to understand the information regarding wireless monitoring or who are unable to provide consent, and (3) patients currently participating in another clinical trial that may be affected by the implementation of wireless monitoring. Patients who have previously participated in clinical trials, those who are currently participating in a clinical trial that the investigator judges will not affect or be affected by this trial, and those who are participating in other observational studies are eligible for this study. If the patient wishes to discontinue participation in the study or dies within 6 months of participation (to be noted separately), they will be considered withdrawn from the study.

Informed consent will be obtained from each participant included in the study. Details of the study protocol are available at ClinicalTrials.gov (study ID: NCT05971225). Patient enrolment started in November 2023 at two centres (40 and 10 patients were enrolled in St. Vincent’s Hospital and Eunpyeong St. Mary’s Hospital, respectively, as of 1 August 2024) after Institutional Review Board approval (VC23DIDI0148; approval date: 11 July 2023) and will continue until October 2025. Follow-up data will be acquired for 24 months after enrolment. We plan to collect the final data in December 2027, and the results of the ReVe study will be published in 2028.

The other six participating centres are obtaining approval from their Institutional Review Boards (IRBs). The IRBs will independently monitor the study protocol and process and conduct audits every 6 months from the enrolment of the first patient. The investigation conforms with the principles outlined in the Declaration of Helsinki and is supported by funding from Biotronik.

### 2.2. Data Collection

The baseline data include patient demographic details, such as gender, date of birth, cardiac device type, implantation date, and indication for the device implantation. They also include health conditions (such as a history of hypertension or heart failure) and information on prescription drugs at enrolment, obtained from electronic medical records.

We determined that a historical group is necessary to compare and analyse patients’ hospitalisation rates and satisfaction before and after the application of RM. Patients in the historical group were diagnosed with heart diseases a considerable time before enrolment. They will be compared to patients in the initiating group to determine whether the hospitalisation rate improves with the application of RM.

The two patient groups in this study are as follows: (1) the historical group consisting of patients who already have CIEDs (implanted after January 2020) and have attended outpatient device check-ups (225 patients planned); (2) the initiating group consisting of patients who will undergo CIED implantation after providing informed consent for participation in this study (225 patients planned). We want to investigate the convenience of and satisfaction with RM applications for patients who have yet to experience RM and obtain information on major adverse cardiac and cerebrovascular events (MACCEs) that occurred before RM was applied. Considering the patients’ average lifespan, analysing MACCEs only in the historical group will cause limitations in the future. Therefore, we will also determine the MACCEs in the initiating group for comparison. After informed consent was provided, the patients or their caregivers received a CardioMessenger Smart transmitter (CM) paired with their CIED. The CM collects information through wireless technology (including therapeutic interventions, arrhythmic events, thoracic impedance information, lead data, and device-related alerts), encodes the collected biometric data, and transmits it to a medical institution. Daily RM will be initiated the day after device pairing. In the case of long business trips or other types of extended travelling, patients are asked to take the CM with them and keep it connected to a charger at night near their bedside.

In this study, we may also use the successor model of the CM family. The package provided to participants will consist of the CM and a power supply module. The CM receives information from the paired CIED once daily, typically at night. The transmitter is used for communication with the CIED, and the data received from the device are encrypted and sent to the company website (www.biotronik-homemonitoring.com/, accessed on 6 February 2025) via the data module. Healthcare providers receive an alert from the website when the device detects a change in the patient’s heart rhythm, identifies issues with the device itself, or prompts a reminder for periodic, overall CIED check-ups.

The ReVe study will be the first prospective multicentre study in South Korea in which the following conditions are fulfilled:

(1) Collecting data from all visits or admissions during the study period, classified as device-related, patient cardiovascular condition-related, or non-cardiovascular condition-related events. These visits will also be categorised as either physician-driven (RM-based) or patient-driven hospital visits.
(2) Analysing MACCEs, such as myocardial infarctions/cerebrovascular accidents, thromboembolic events, exacerbation of heart failure, admissions due to cardiac causes, cardiac-related mortality (as a proportion of overall mortality), and other cardiac events.
(3) Collecting and analysing data on RM variables weekly, including thoracic impedance and lead sensing, to elucidate their correlation with disease progression, admission, and mortality.
(4) Evaluating the staffing/time requirements for RM using electronic medical records (log-in and log-out times are recorded).
(5) Analysing and comparing patient satisfaction questionnaires after RM every 6 months (Table 1) via telephone or during office visits (between the historical and initiating groups). The questionnaire evaluates the efficacy of RM and the perceived quality of care due to RM. It contains nine questions with five answer categories each (strongly agree, agree, neutral, disagree, and strongly disagree).

**Figure 1:**
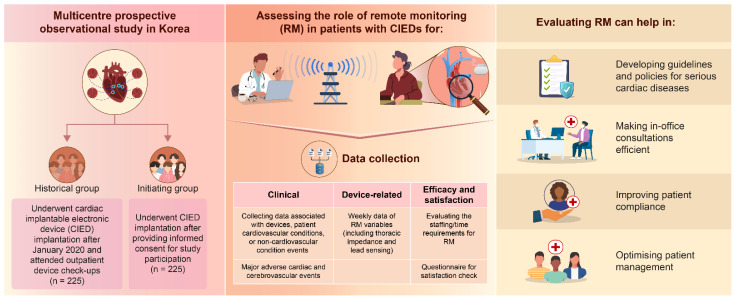
ReVe study protocol.

Questions (1) to (3) contain the primary outcomes, and (4) and (5) include the secondary outcomes. These are summarised in Figure 1.

Patients with CIEDs, except those on warfarin or those with other relevant medical issues (followed up on other clinic consultation schedules), are scheduled for routine clinical follow-ups every 3–6 months. Patient satisfaction surveys are scheduled every 6 months to assess changes in satisfaction levels over time and explore varying trends across device types (‘Survey on telehealth patient experience’ by YouMi Hwang, 2023 [15], modified Korean version 1.3; Table 1). The study is summarised in Figure 1, and the process of the study is also available in Supplementary Table S1.

The primary endpoint will be any MACCE after RM (cardiovascular-related death and hospital admissions related to pre-existing cardiovascular disease), defining variables that are predictive of MACCE and unexpected hospital visits during RM. The secondary endpoints will be patient satisfaction with the time, cost, and quality of care after adopting RM (determined via questionnaires on a 6-month basis) and the staffing/time requirements during RM (measured using electronic medical records).

All files and data generated during the study are stored in a safety deposit box or as copied files secured with passwords accessible only to the investigators. Because this study includes personal identifiers and sensitive information, the data will not be openly accessible. Anonymised study data will be made available to researchers upon reasonable request after the study results are published.

### 2.3. Statistical Analysis

We will compare outcomes before and after RM via paired *t*-tests in the historical group. We will compare the clinical-, RM-, and device-related variables via independent samples *t*-test between the historic and initiating RM groups. Tests for normality will be provided. If data is non-normal, then *t*-tests and means/standard deviations should be avoided, and Mann–Whitney/Wilcoxon and medians/interquartile ranges should be preferred. For the *t*-tests, we will reject the null hypothesis when the two-sided *p*-value is less than 5%. For the analysis of patient baseline data, continuous variables will be presented as means and standard deviations and analysed using *t*-tests or Wilcoxon rank-sum tests. Categorical variables will be presented as frequencies and percentages. For the analysis of satisfaction with the survey, paired *t*-tests (parametric test) or Wilcoxon signed-rank tests (non-parametric test) will be performed. For surveys conducted only after monitoring, ordinal variables will be analysed using the Mann–Whitney U test, and continuous variables will be analysed using chi-square tests. For patients not completing the follow-up, censoring will be based on the last data collected. Cumulative event probabilities will be estimated with adverse health outcomes, such as patient deaths, inpatient admissions, and MACCEs. Hazard ratios and 95% confidence intervals will be generated with Cox proportional hazards models. For subgroup analyses, the interaction term between the prespecified groups, based on the type of underlying heart disease and LVEF, will be evaluated for the primary outcome. Because of the interim analyses, we will adopt the O’Brien–Fleming method for statistical corrections. For all analyses, we will use two statistical software packages: Stata, version 18 (StataCorp LLC, College Station, TX, USA), and R, version 4.05 (R Foundation for Statistical Computing, Vienna, Austria).

## 3. Discussion

This study will provide evidence for the development of guidelines and policies for RM of patients suffering from serious cardiac diseases. It may also assist in establishing insurance standards for RM in South Korea. Patients with CIEDs need regular follow-ups for their underlying cardiac conditions, alongside planned check-ups for the implanted devices. Although the use and provision of expensive and precise medical technologies, procedures, and drugs to many people at low prices are desirable, the number of patients is too large compared to that of medical staff who offer such services. Thus, time and physical constraints limit the provision of appropriate services. As physicians have an up-to-date understanding of patient health information before the visit, RM adoption can improve the efficiency of short, in-office consultations, allowing them to provide appropriate services. The perspectives of patients with CIEDs and the physicians may differ, as patients desire adequate attention and care. Therefore, the abovementioned factors may lead to patient and/or physician dissatisfaction and inadequate knowledge of CIED recipients regarding their disease status. This may result in poor patient compliance regarding care and self-administered treatments [17]. The number of CIED recipients is gradually increasing owing to the ageing population, which may exacerbate compliance problems and result in poor clinical outcomes. The need for RM of CIEDs is more important for individualised and optimised patient management than ever. To effectively utilise RM, cooperation from patients and their guardians and a sufficient understanding and education regarding RM and their disease are essential. Patient awareness and collaboration may contribute to their satisfaction and improve clinical outcomes.

The workload of RM-related personnel and establishing adequate fees for their medical services remain unsolved [15,18]. Consensus guidelines are not feasible because of the differences in healthcare systems worldwide [19,20,21,22,23,24,25,26]. In a national insurance system such as that in South Korea, evaluating specialised personnel and adequate monitoring fees is necessary to enable the widespread adoption of RM. As monitoring is crucial for patients with structural heart diseases, we believe that the ReVe study can serve as a basis for preparing the professional manpower needed for RM, provide evidence for the time required for RM, and establish an appropriate medical fee system. Countries with similar healthcare systems can work together toward solutions. However, RM-related personnel education and preparation are crucial to fully utilise RM in medical services. This study is anticipated to assist in establishing guidelines for patients with CIEDs and underlying structural heart diseases who need more dedicated and tailored management. Further, this study will provide sound evidence for developing healthcare policies and determining suitable medical costs and RM-related personnel requirements in South Korea and other countries with similar national health insurance systems. The results of the ReVe study will improve the prognosis and safety of patients with severe heart disease and allow for assessing the resulting demand for specialised personnel and appropriate medical costs (The ReVe study is registered on ClinicalTrials.gov under the study ID NCT05971225).

## Figures and Tables

**Table 1:** Survey on patient satisfaction with remote monitoring (modified Korean version by YouMi Hwang).


Question	Strongly Agree	Agree	Neutral	Disagree	Strongly Disagree
I experienced much overall discomfort owing to remote monitoring					
2. I feel that my health and the CIED were better cared for with remote monitoring than with conventional in-office visits					
3. Using CardioMessenger was difficult					
4. Using CardioMessenger caused an inconvenience in my daily life					
5. Remote monitoring has helped me manage my health					
6. I am satisfied with the use of remote monitoring					
7. Remote monitoring has advantages in terms of time and cost compared to visiting the hospital					
8. I want to continue remote monitoring					
9. I would like to recommend remote monitoring to other patients with CIEDs					

CIED, cardiac implantable electronic device.

## Data Availability

Data used in this study are and will be available from the corresponding author (S.-W.J.) upon reasonable request to bona fide researchers.

## Supplementary Materials

The following supporting information can be downloaded at https://www.mdpi.com/article/10.3390/jcm14041150/s1, Table S1: Summarized study process and checklist.
